# Eco-friendly synthesis and antimicrobial activities of some 1-phenyl-3(5-bromothiophen-2-yl)-5-(substituted phenyl)-2-pyrazolines

**DOI:** 10.1186/2191-2858-2-20

**Published:** 2012-06-11

**Authors:** Ramalingam Sasikala, Kannan Thirumurthy, Perumal Mayavel, Ganesamoorthy Thirunarayanan

**Affiliations:** 1 Department of Chemistry, Annamalai University, Annamalai, Nagar 608 002, India

**Keywords:** 1-phenyl-3(5-bromothiophen-2-yl)-5-(substituted phenyl)-2-pyrazolines, Fly ash:H_2_SO_4_, Environmentally benign reaction, IR and NMR spectra, Antimicrobial activities

## Abstract

**Background:**

Green catalyst fly ash: H_2_SO_4_ was prepared by mixing fly ash and sulphuric acid. Microwave irradiations are applied for solid phase cyclization of 5-bromo-2-thienyl chalcones and phenyl hydrazine hydrate in the presence of fly ash: H_2_SO_4_ yields, 1-phenyl-3(5-bromothiophen-2-yl)-5-(substituted phenyl)-2-pyrazolines. These pyrazolines were characterized by their physical constants and spectral data. The antimicrobial activities of all synthesized pyrazolines have been studied.

**Results:**

Scanning electron microscopy (SEM) analysis shows the morphology changes between fly ash and the catalyst fly ash: H_2_SO_4_. The SEM photographs with the scale of 1 and 50 μm show the fly-ash particle is corroded by H_2_SO_4_ (indicated by arrow mark), and this may be due to dissolution of fly ash by H_2_SO_4_. The yields of 1-phenyl-3(5-bromothiophen-2-yl)-5-(substituted phenyl)-2-pyrazolines is more than 75% using this catalyst under microwave heating. All pyrazolines showed moderate activities against antimicrobial strains.

**Conclusion:**

We have developed an efficient catalytic method for synthesis of 1-phenyl-3(5-bromothiophen-2-yl)-5-(substituted phenyl)-2-pyrazolines by solid phase cyclization using a solvent-free environmentally greener catalyst fly ash: H_2_SO_4_ under microwave irradiation between aryl chalcones and hydrazine hydrate. This reaction protocol offers a simple, economical, environment friendly, non-hazardous, easier work-up procedure, and good yields. All synthesized pyrazoline derivatives showed moderate antimicrobial activities against bacterial and fungal strains.

## Background

Pyrazolines are well-known important nitrogen containing five membered heterocyclic bioorganic molecules. The pyrazoline ring protons were bonded with carbon atoms on a spatially different environment. These pyrazolines are used widely in the current decades due to their various biological and pharmacological activities [1]. The α,β-unsaturated ketones can play the role of versatile precursors in the synthesis of the corresponding pyrazolines [2-7]. Numerous methods have been reported for the preparation of pyrazoline compounds. Fischer and Knoevenagel in the nineteenth century studied the reaction of α,β-unsaturated aldehydes and ketones with phenyl hydrazine in acetic acid by refluxing, which became one of the most popular methods for the preparation of 2-pyrazolines [8]. In 1998, Powers et al. [9] have reported the reaction of chalcones with phenyl hydrazine hydrochloride in the presence of sodium hydroxide and absolute ethanol at 70°C, where the longer reaction time is the disadvantage of the reaction. K_2_CO_3_-mediated microwave irradiation has been shown to be an efficient method for the synthesis of pyrazolines [10]. The regioselective formation of pyrazolines has been synthesized by the reaction of substituted hydrazine with α,β-unsaturated ketones [11,12]. Recently, many organic reactions in aqueous media have been described in the literature [13]. In 2007, Li et al. [14] have synthesized 1,3,5-triaryl-2-pyrazoline with chalcones and phenyl hydrazine hydrochloride in sodium acetate-acetic acid aqueous solution under ultrasound irradiation. Pyrazolines have been exhibiting various pharmacological activities, such as analgesic [15], anti-inflammatory [16,17], antimicrobial [18,19], anti-amoebic [20,21], antitubercular [22,23], hypoglycemic [24], anticoagulant [25], antidepressant [26-28], pesticide [29], fungicide [30], antibacterial [31], and anticonvulsant activities [32]. Recent report shows some new pyrazoline-substituted thiazolone-based compounds that exhibit anticancer activity [33]. Apart from biological activities, pyrazolines are also extensively used as synthons in organic synthesis [34-36], as optical brightening agents for textiles, paper, and fabrics, and as a hole-conveying medium in photoconductive materials [37-41]. In this present study, the authors have taken efforts to synthesize a series of 1-phenyl-3(5-bromothiophen-2-yl)-5-(substituted phenyl)-2-pyrazolines from 5-bromo-2-thienyl chalcones and phenyl hydrazine hydrate in presence of fly ash:H_2_SO_4_. These pyrazolines were characterized by their physical constants and spectral data. The antimicrobial activities of all synthesized pyrazolines have been studied.

## Methods

### Materials

All chemicals used were purchased from Sigma-Aldrich Corporation (St. Louis, MO, USA) and E-Merck chemical company (Merck Limited, Mumbai, India) Melting points of all pyrazolines have been determined in open glass capillaries on Mettler FP51 melting point apparatus (Mettler-Toledo India Private Limited, Mumbai, India) and are uncorrected. Infrared spectra (KBr, 4,000 to 400 cm^−1^) have been recorded on AVATAR-300 Fourier transform spectrophotometer (Thermo Nicolet, USA). A BRUKER AMX-500 NMR spectrometer (BRUKER AXS GMBH, Karlsruhe, Germany) operating at 500 MHz has been utilized for recording ^1^ H spectra and 125.75 MHz for ^13^C spectra in CDCl_3_ solvent using TMS as internal standard. Electron impact (70 eV) and chemical ionization mode FAB^+^ mass spectra have been recorded in VARIAN-SATURN 2200 GC-MS spectrometer (Varian Medical Systems, Palo Alto, CA, USA).

## Results and discussion

Fly ash is a waste air pollutant, and it has many chemical species [10,33], such as SiO_2_, Fe_2_O_3_, Al_2_O_3_, CaO, and MgO, and insoluble residues. The waste fly ash is converted into useful catalyst fly ash: H_2_SO_4_ by mixing fly ash and sulphuric acid. The sulphuric acid-sulphate ion group and chemical species present in the fly ash have enhanced catalytic activity. During the course of the reactions, these species are responsible for the promoting effects on cyclization between the chalcones and hydrazine hydrate leading to the formation of pyrazolines. In these experiments, the products were isolated, and the catalyst was washed with ethyl acetate, heated to 100°C, and was then reusable for further five run reactions. There was no appreciable change in the percentage of yield of pyrazolines. In this protocol, the reaction gave better yields of the pyrazolines during the condensation without any environmental discharge. The analytical and mass spectral data are presented in Table 1. The infrared (IR) and nuclear magnetic resonance (NMR) spectral data of unknown pyrazolines are presented in Tables 2, and 3.

**Table 1:** Analytical and mass spectral data of 1-phenyl-3(5-bromothiophen-2-yl)-5-(substituted phenyl)-2-pyrazolines

**Entry**	**Substituent**	**MF**	**FW (dalton)**	**Yield (%)**	**M.p. (°C)**	**Mass (m/z)**
1	H	C_20_H_17_BrN_2_	365	85	152-153	365[M^+^], 367[M^+2^], 287, 285, 221, 209, 142, 79, 77, 68, 65, 41, 28, 14
2	4-Br	C_20_H_16_Br_2_N_2_	444	83	148-150	444[M^+^], 446[M^+2^], 448[M^+4^], 365, 363, 229, 222, 201, 155, 142, 77, 68, 41, 28, 14.
3	2-Cl	C_20_H_17_BrClN_2_	399	75	142-144	399[M^+^], 401[M^+2^], 403[M^+4^], 363, 321, 319, 287, 225, 209, 142, 111, 77, 68, 65, 41, 28, 35, 14
4	4-Cl	C_20_H_17_BrClN_2_	399	79	147-149	399[M^+^], 401[M^+2^], 403[M^+4^], 363, 321, 287, 225, 209, 142 77, 65, 41, 28, 14
5	3, 4-(OCH_3_)_2_	C_22_H_21_BrN_2_O_2_	425	85	140-142	425[M^+^], 427[M^+2^], 393, 363, 347, 287, 251, 142, 134, 107, 95, 78, 65, 41, 28, 14
6	4-I	C_20_H_17_BrIN_2_	491	76	146-148	491[M^+^], 493[M^+2^], 495[M^+4^], 412, 363, 347, 287, 269, 229, 203, 142, 126, 77, 42, 28, 14
7	4-(OCH_3_)	C_21_H_19_BrN_2_O	395	85	128-130	395[M^+^], 397[M^+2^], 363, 317, 287, 210, 142, 105, 77, 41, 14
8	4-CH_3_	C_21_H_19_BrN_2_	379	83	148-149	379[M^+^], 381[M^+2^], 365, 287, 235, 210, 142, 91, 77, 41, 14

FW, formula weight; MF, molecular formula; M.p., melting point; m/z, mass-to-change ratio.

**Table 2:** IR and NMR spectral data of 1-phenyl-3(5-bromothiophen-2-yl)-5-(substituted phenyl)-2-pyrazolines

**Entry X**		**Infrared bands (ν cm**^**−1**^**)**					^**1 **^**H chemical shifts *δ *(ppm)**					**Entry X**		^**13 **^**C chemical shifts *δ *(ppm)**				
		**C = N**	**C-S**	**C-Br**	**Ar-C and Alip-C**	**Subst.**	**Ha (1 H, *dd*)**	**Hb (1 H, *dd*)**	**Hc (1 H, *dd*)**	**Ar-H**	**Subst.**			**C**_**3**_	**C**_**4**_	**C**_**5**_	**Ar-C**	**Subst.**
1	H	1,593	679	562	3028-2854	-	3.06	3.77	5.25	6.71-7.34	-	1	H	155.60	43.66	64.68	111.77-146.521	-
							*J* = 24 Hz	*J* = 29 Hz	*J* = 19 Hz	(12 H, m)								
2	4-Br	1,596	685	5,621	3089-2852	-	3.07	3.82	5.26	6.70-7.51	-	2	4-Br	155.56	43.45	64.10	112.13-144.13	-
							*J* = 30 Hz	*J* = 28 Hz	*J* = 24 Hz	(11 H, m)								
3	2-Cl	1,595	690	566	3065-2852	-	3.01	3.93	5.66	6.77-7.48	-	3	2-Cl	157.50	42.08	61.50	113.28-143.99	-
							*J* = 23 Hz	*J* = 29 Hz	*J* = 19 Hz	(11 H, m)								
4	4-Cl	1,594	688	5,31	3046-2852	-	3.10	3.80	5.28	6.75-7.35	-	4	4-Cl	155.82	43.70	64.75	113.52-144.40	-
							*J* = 24 Hz	*J* = 29 Hz	*J* = 19 Hz	(11 H, m)								
5	3,4-(OCH_3_)_2_	1,594	678	569	3073-2873	1,254	3.08	3.76	5.17	6.75-7.36	3.83, 3.87	5	3,4-(OCH_3_)_2_	155.63	43.81	64.89	111.68-149.74	55.94, 55.98
							*J* = 25 Hz	*J* = 30 Hz	*J* = 19 Hz	(11 H, m)								
6	4-I	1,595	680	531	3090-2852	-	3.04	3.78	5.21	6.74-6.69	-	6	4-I	156.11	43.52	64.21	93.16-143.45	-
							*J* = 24 Hz	*J* = 29 Hz	*J* = 20 Hz	(11 H, m)								
7	4-OCH_3_	1,596	670	553	3095-2852	1,249	3.07	3.77	5.24	6.75-7.28	3.80	7	4-OCH_3_	155.81	43.75	64.29	113.57-159.13	55.28
							*J* = 25 Hz	*J* = 31 Hz	*J* = 18 Hz	(11 H, m)								
8	4-CH_3_	1,594	680	546	3095-2854	-	3.07	3.77	5.25	6.71-7.34	2.31	8	4-CH_3_	156.10	43.72	64.47	111.68-146.48	21.13
							*J* = 24 Hz	*J* = 28 Hz	*J* = 19 Hz	(11 H, m)								

**Table 3:** **The HOMOCOSY and HSQC data *δ *(parts per million) of 1-phenyl-3(5-bromothiophen-2-yl)-5-(phenyl)-2-pyrazoline (1)**

**HOMOCOSY**						**HSQC**				
**Proton**	**Carbon (ppm)**	***δ *H**_**a**_**at C**_**4**_**3.06**	***δ *H**_**b**_**at C**_**4**_**3.77**	***δ *H**_**c**_**at C**_**5**_**5.25**	***δ *Ar-H 6.71-7.34**	**Proton**	**Carbon (ppm)**	***δ *C**_**4**_**43.66**	***δ *C**_**5**_**64.68**	***δ *Ar-H 111.77-46.52**
*δ* H_a_ at C_4_	3.06	-	Bonded	Bonded	-	*δ* Ha at C4	3.06	Bonded	-	-
*δ* H_b_ at C_4_	3.77	Bonded	-	Bonded	-	*δ* Hb at C4	3.77	Bonded	-	-
*δ* H_c_ at C_5_	5.25	Bonded	Bonded	-	-	*δ* Hc at C5	5.25	-	Bonded	-
*δ* Ar-H	6.71-7.34	-	-	-	Bonded	*δ* Ar-H	6.71-7.34	-	-	Bonded

HOMOCOSY, homonuclear correlation spectroscopy; HSQC, heteronuclear single quantum correlation.

**Table 4:** The antibacterial and antifungal activities of 1-phenyl-3(5-bromothiophen-2-yl)-5-(substitutedphenyl)-2-pyrazolines by disc diffusion method

**Antibacterial activities**							**Antifungal activities**				
**Entry X**		**Zone of inhibition (mm)**					**Entry X**	**Zone of inhibition (mm)**			
		***S. aureus***	***E. coli***	***K. pneumoniae***	***S. typhi***	***Pseudomonas spp.***		***C. albicans***	***Mucor spp.***	***Rhizopus spp.***	***A. niger***
1	H	10	15	20	15	15	1	15	15	15	15
2	4-Br	10	15	20	20	17	2	20	20	20	20
3	2-Cl	15	15	15	5	5	3	15	15	15	15
4	4-Cl	3	10	15	15	15	4	15	15	15	15
5	3,4-(OCH_3_)_2_	17	17	15	17	20	5	17	17	17	17
6	4-I	15	17	20	17	10	6	17	17	17	17
7	4-OCH_3_	15	17	20	17	22	7	20	20	20	20
8	4-CH_3_	15	10	15	5	5	8	20	15	15	20
Cip	-	17	17	20	22	15	Ket	15	15	15	15

Cip, ciprofloxacin; Ket, ketoconazole.

### Infrared spectral study

In the IR spectra of synthesized pyrazolines, the stretching frequency at 1,593 to 1,596 cm^−1^ is assigned to C = N. The frequency at 678 to 688 cm^−1^ is due to the C-S group, and a band at 531 to 569 cm^−1^ is assigned to the C-Br stretching frequency. A collection of medium bands observed in the region 3,028 to 2,854 cm^−1^ is attributed to C-H stretching vibrations of the aliphatic and aromatic groups. These IR bands are supporting evidences for the formation of pyrazolines. The assigned spectral bands of pyrazolines are presented in Table 2.

### NMR spectral study

#### ^1^ H NMR spectra

In ^1^ H NMR spectrum of pyrazolines, the doublet of doublet at *δ* 3.01 to 3.10 with coupling constants *J*_1_ = 7.5 Hz and *J*_2_ = 17 Hz is assigned to H_a_ proton of C_4_. The doublet of doublet at 3.76 to 3.93 ppm with coupling constants *J*_1_ = 12.5 Hz and *J*_2_ = 17 Hz is assigned to H_b_ proton of C_4_. Similarly, the doublet of doublet at 5.17 to 5.66 ppm with coupling constants *J*_1_ = 7.5 Hz and *J*_2_ = 12.5 Hz is assigned to H_c_ proton of C_5_. The aromatic protons appeared in the range of 6.71 to 7.34 ppm. These proton chemical shift (part per million) values were supported for formation of pyrazolines and are presented in Table 2.

#### ^13^ C NMR spectra

In ^13^ C NMR spectrum of pyrazolines, the signals appear in the range 111.77 to 146.52 ppm are due to the aromatic carbons. The signal at downfield region 155.60 ppm are assigned to C = N carbon. Two signals that appeared in the lower frequency region at 43.66 and 64.68 ppm are assigned to the methylene and methyn carbons at C_4_ and C_5_, respectively. The assigned ^13^ C chemical shift values of all pyrazolines are furnished in Table 2.

#### HOMOCOSY spectra

**Figure 1:**
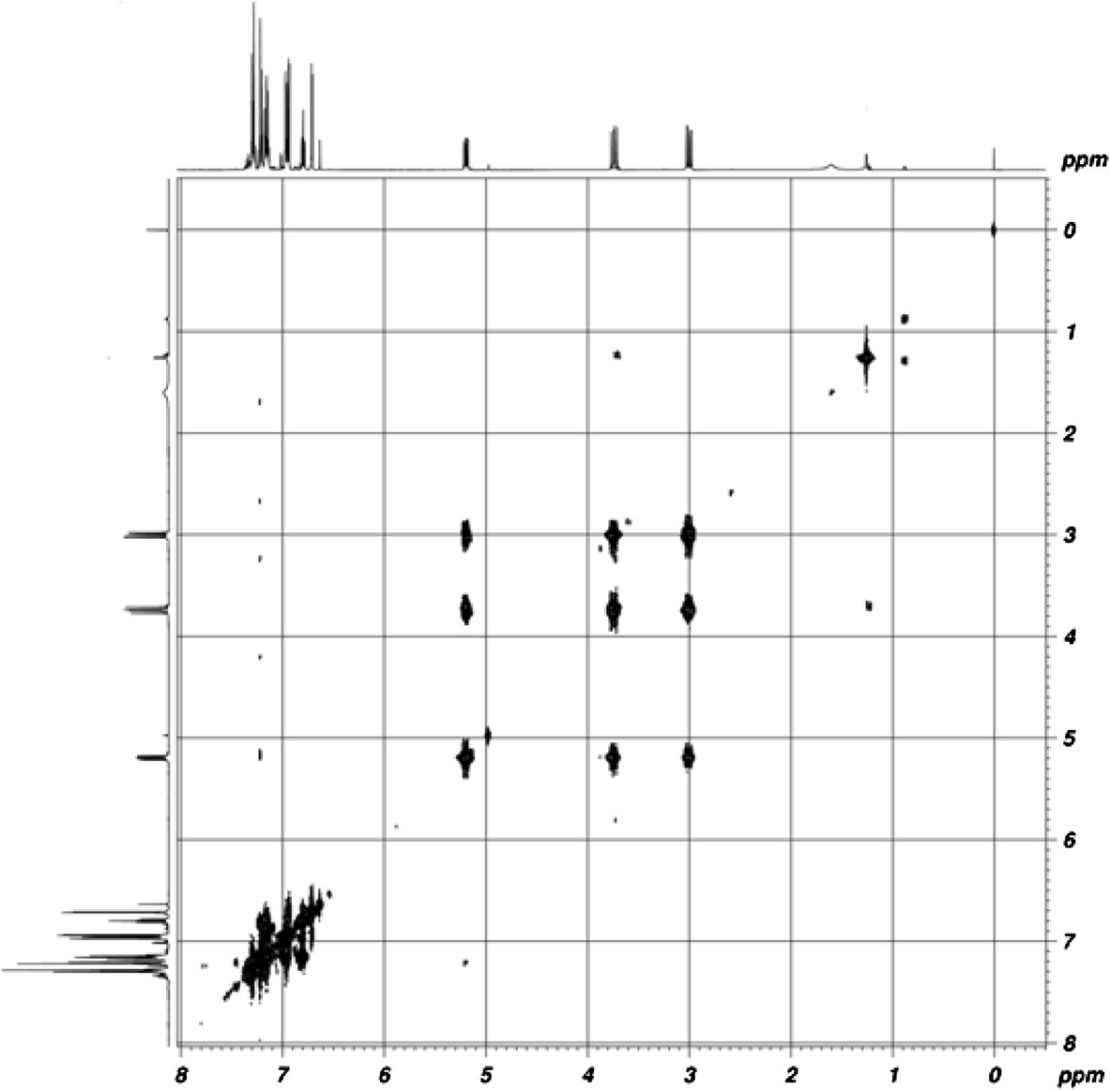
HOMOCOSY spectrum of 1-phenyl-3(5-bromothiophen-2-yl)-5-(phenyl)-2-pyrazoline (1).

In the homonuclear correlation spectroscopy (HOMOCOSY) spectrum of pyrazolines, the possible correlations are furnished in Table 3. The ^1^ H-^1^ H COSY spectrum of parent compound (entry 1) is shown in Figure 1. Both H_a_ (3.06 ppm) and H_b_ (3.77 ppm) protons at C_4_ show a strong cross peak with the signal at 5.25 ppm. This suggests that the doublet of doublet at 5.25 ppm is due to the H_c_ proton of C_4_. Based on this analysis, the proton chemical shifts (parts per million) of other pyrazolines were assigned and confirmed.

#### HETCOSY spectra

**Figure 2:**
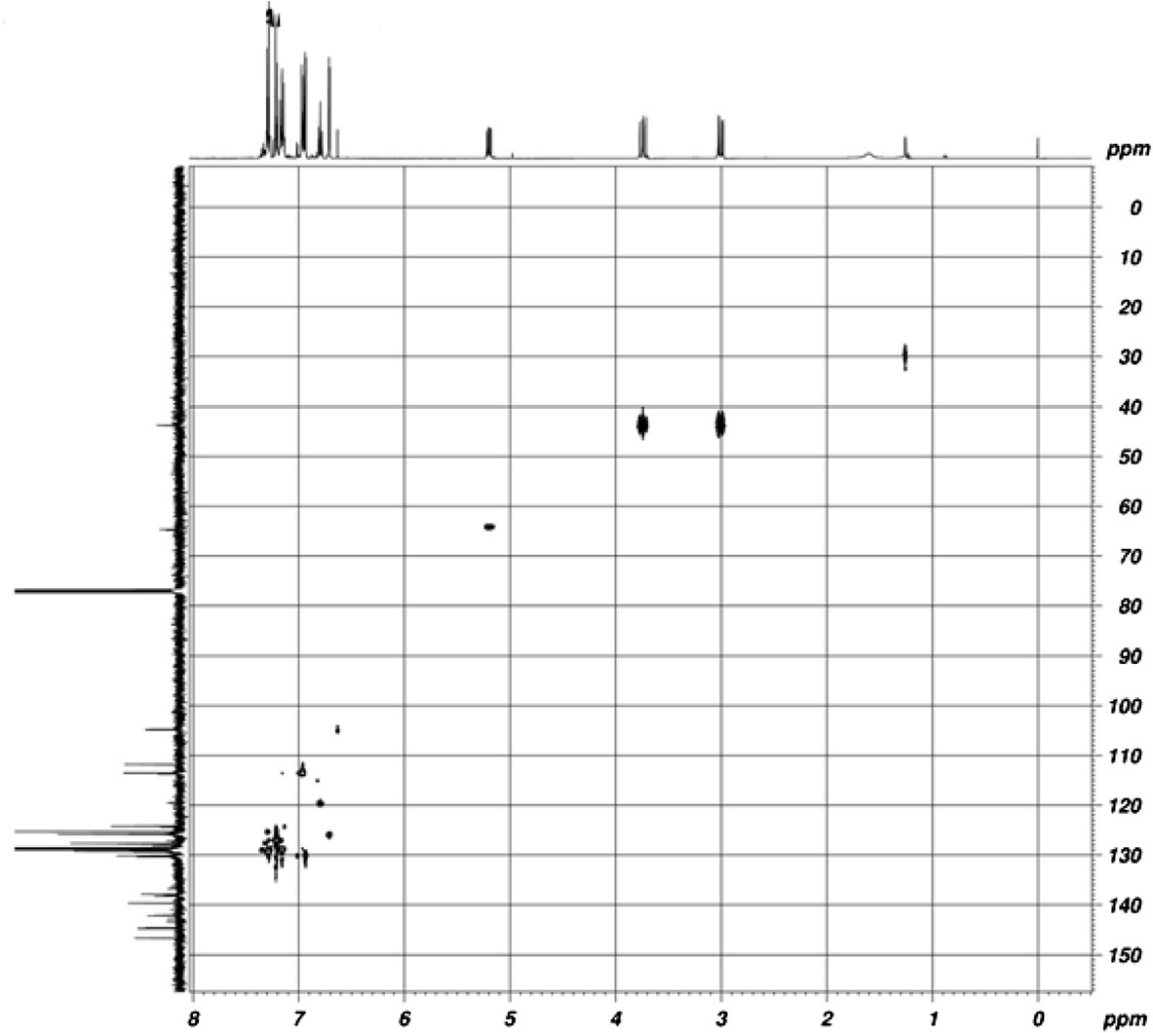
HSQC spectrum of 1-phenyl-3(5-bromothiophen-2-yl)-5-(phenyl)-2-pyrazoline (1).

The HSQC (heteronuclear single quantum correlation) spectrum of synthesized pyrazoline (entry 1) is shown in Figure 2, and their correlations are listed in Table 3. The H_a_ and H_b_ protons at C_4_ show a cross peak with the carbon signal at 43.66 ppm, which confirms that this carbon signal is due to C_4_ carbon. Similarly, a doublet of doublet at 5.25 ppm is having a strong correlation with the carbon resonance at 64.68 ppm. From this, it is inferred that the carbon signal is due to C_5_ carbon. Based on this analysis, the carbon chemical shifts (parts per million) of other pyrazolines were assigned and confirmed.

### Antimicrobial activities

#### Antibacterial activity

*Staphylococcus aureus* was taken as gram positive strain, and *Escherichia coli*, *Klebsiella pneumoniae*, *Salmonella typhi*, and *Pseudomonas* species were taken as gram negative strains; they have been used for the present study.

#### Determination of antibacterial activity by disc-diffusion method

Nutrient agar plates were prepared under sterilized conditions and incubated overnight to detect contamination. About 0.2 mL of working stock culture was transferred into separate nutrient broth and spread thoroughly using a glass spreader. Whatman number 1 discs (6 mm in diameter) were impregnated with the test compounds dissolved in DMSO (200 μg/mL) for about half an hour. Commercially available drug disc (ciprofloxacin 10 mg/disc) was used as positive reference standard. Negative control was also prepared by impregnating the disc of same size in dimethyl sulfoxide (DMSO) solvent. The plates were then incubated overnight for 18 to 24 h. Antibacterial activity was evaluated by measuring the zone of inhibition against the test organism.

#### Determination of minimum inhibitory concentration of test compounds using twofold serial dilution method

Testing was done in the seeded broth (10^−6^ to 10^−7^ cfu/mL). The test compounds were taken at different concentrations ranging from 200, 100, 50, 25, 12.5, 6.25, 3.13, 1.56, 0.78, and to 0.39 μg/mL for finding the minimum inhibitory concentration (MIC) by using seeded broth as diluent. Similarly, the standard solution of ciprofloxacin drug prepared at the concentrations of 200, 100, 50, 25.5, 6.25, 3.13, 1.56, 0.78, and 0.39 μg/mL of sterile distilled water and DMSO were maintained throughout the experiment simultaneously as control.

The study involves a series of 10 assay tubes for the test compounds against each strain. In the first assay tube, 1.6 mL of seeded broth was transferred, and 0.4 mL of the test solution was added, followed by mixing it thoroughly to obtain a concentration of 200 μg/mL. To the remaining nine assay tubes, 1 mL of seeded broth was transferred, and then, from the first assay tube, per milliliter of the content was pipetted out and added into the second assay tube, followed by mixing thoroughly. This type of dilution was repeated up to the 10th assay tube serially. The same procedure was followed for standard drugs. Duplicates were also maintained; these were done under aseptic conditions.

The racks of assay tubes were placed inside the incubator at 37 ± 1°C for 24 h. After the incubation period, the assay tube concentrations were again streaked into the nutrient agar plate due to turbidity of the drug microorganism mixture. The lowest concentration of the test compounds, which caused apparently a complete inhibition of growth of organisms, was taken as the MIC. The solvent control tube was also observed to find whether there was any inhibitory action. The sterile distilled water and DMSO did not show any inhibition.

**Figure 3:**
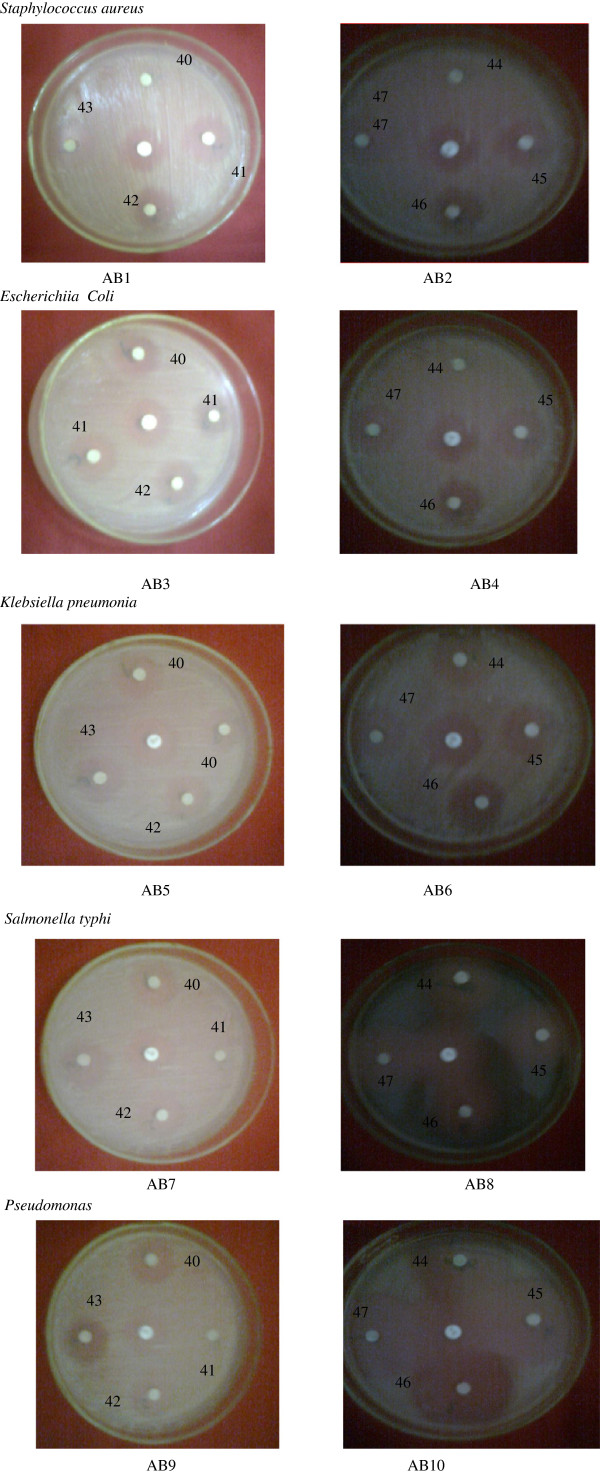
Antibacterial activities by zone of inhibition of 1-phenyl-3(5-bromothiophen-2-yl)-5-(substituted phenyl)-2-pyrazoline petri dishes (AB1 to AB10).

The antimicrobial activity of all the synthesized pyrazolines (entries 1 to 8) were examined by disc diffusion and two fold serial dilution methods. Bacterial strains, *viz. S. aureus*, *E. coli*, *K. pneumonia*, *S. typhi*, and *Pseudomonas* species, and fungal strains, *viz. Candida albicans*, *Mucor* species, *Rhizopus* species, *Aspergillus niger*. In the present study, DMSO is used as control, while ciprofloxacin and ketoconazole are used as standards for bacterial and fungal strains, respectively. The zone of inhibition and MIC values of compounds (entries 1 to 8) against both the tested bacterial strains are given in Table 4. The representative photographs of disc diffusion and serial dilution methods are depicted in Figure 3.

The antibacterial activity of all pyrazolines produced a maximum zone of inhibition against all the bacterial strains except compounds 3 and 4 against *S. typhi* and *Pseudomonas* spp., compounds 4 against *S. aureus*, and compounds 7 and 8 against *Pseudomonas* spp. which showed maximum zone of inhibition than the standard (ciprofloxacin).

Antibacterial activity of all synthesized pyrazolines was measured by serial dilution method, and the MICs are presented in Table 5. From Table 5, compounds 1 to 8 showed the growth inhibitory concentration against the tested organism fall in the range of 1.5 to 200 μg/mL. However, compounds 1 to 4 showed the inhibition against all bacterial strains in the range from 25 to 100 μg/mL. The rest of the compounds are more effective against all bacterial strain MICs at 1.5 to 25 μg/mL.

**Table 5:** The antibacterial and antifungal activities of 1-phenyl-3(5-bromothiophen-2-yl)-5-(substituted phenyl)-2-pyrazolines by serial dilution method

**Antibacterial activities**							**Antifungal activities**				
**Entry X**		**MIC (μg/mL)**					**Entry X**	**MIC (μg/mL)**			
		***S. aureus***	***E. coli***	***K. pneumoniae***	***S. typhi***	***Pseudomonas spp.***		***C. albicans***	***Mucor spp.***	***Rhizopus spp.***	***A. niger***
1	H	25	50	12.5	50	50	1	50	50	50	50
2	4-Br	50	25	3.13	3.13	6.25	2	3.13	3.13	3.13	3.13
3	2-Cl	25	25	25	100	100	3	25	25	25	25
4	4-Cl	200	50	25	25	25	4	25	25	25	3.13
5	3,4-(OCH_3_)_2_	6.25	6.25	12.5	6.25	3.13	5	6.25	6.25	6.25	6.25
6	4-I	25	6.25	3.13	6.25	50	6	6.25	6.25	6.25	6.25
7	4-OCH_3_	12.5	6.25	3.13	6.25	1.5	7	3.13	3.13	3.13	3.13
8	4-CH_3_	50	100	50	200	200	8	50	50	50	50
Cip	-	6.25	6.25	3.13	1.5	12.5	Ket	12.5	12.5	12.5	12.5

Cip, ciprofloxacin; Ket, ketoconazole.

#### Antifungal activity

The following fungal strains *C. albicans*, *Mucor* spp, *Rhizopus* spp, and *A. niger* were used for the present study. Sabouraud dextrose agar (SDA) medium was used for the growth of fungi, and testing was done in Sabouraud dextrose broth (SDB) medium.

The subculture and the viable count were carried out by the same procedure as done in antibacterial studies except for the temperature which should be maintained at 28 ± 1°C for about 72 h. Similarly, for the disc diffusion method, the petri dishes were incubated at 28 ± 1°C for about 72 h. The same concentration of the test compound, solvent (DMSO), and ketoconazole (standard) prepared previously were used for the antifungal studies.

**Figure 4:**
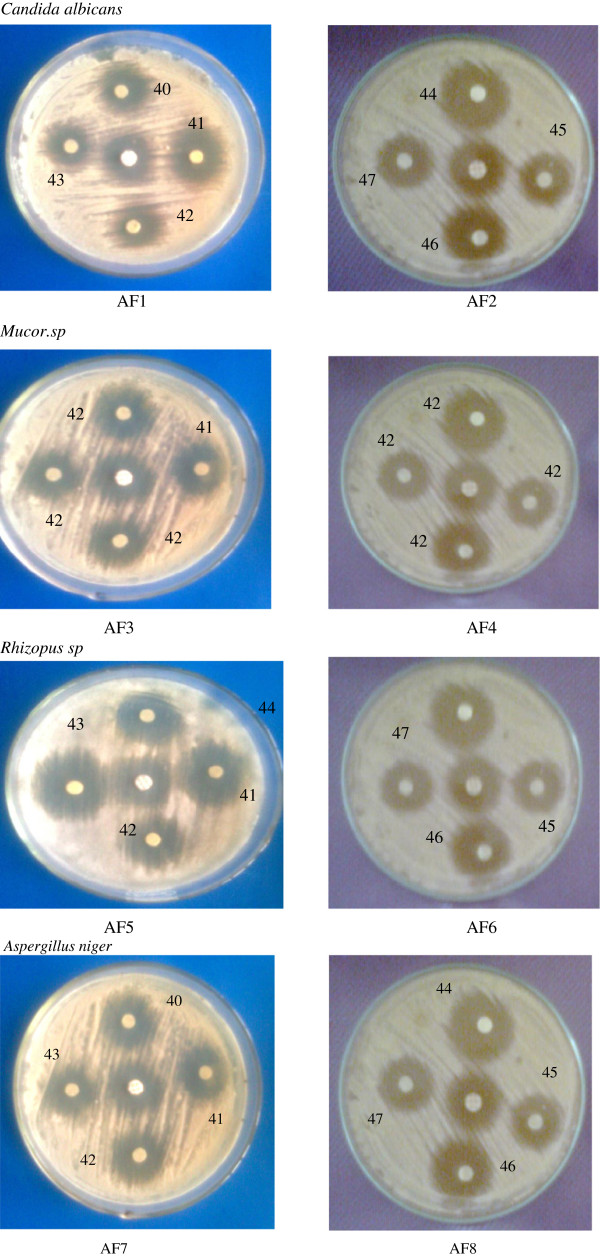
Antifungal activities of spectrum of 1-phenyl-3(5-bromothiophen-2-yl)-5-(substituted phenyl)-2-pyrazoline pretri dishes (AF1 to AF8).

The antifungal activities of synthesized pyrazolines 1 to 4 exhibited a similar inhibition activity as that of the standard (15 mm) against all fungal strains, whereas pyrazoline 2 exhibited against *C. albicans* and *A. niger* (20 mm), and pyrazoline 5 to 8 against all the fungal strains showed maximum zone of inhibition (17 to 20 mm) than the standards (15 mm, ketoconazole). The measured antifungal activities of pyrazolines are presented in Table 4. The representative photographs of disc diffusion methods are depicted in Figure 4.

The antifungal activities of all synthesized pyrazolines were measured by serial dilution method. Among the compounds under study, compounds 1 to 8 were found to be effective against all the fungal strain MICs at 3.13 to 6.25 μg/mL. MIC values of compounds 1 to 4 against all the tested fungal strains are in the range of 25 to 50 μg/mL except for compound 2 against *C. albicans* (12.5 μg/mL) and compound 4 against *A. niger* (3.13 μg/mL). The measured antifungal activities of all compounds are presented in Table 5.

### Comparison of potency of compounds 1 to 8 with standard drugs against bacterial and fungal strains from serial dilution method

In order to understand the results of serial dilution method, the potency of synthesized compounds 1 to 8 against tested bacterial and fungal strains are calculated with respect to the reference (standards) using Equation 1:

(1) $$\begin{array}{l} \text{Potency}\;\left(\%\right)=\frac{\text{M IC}\;\left(\text{mg}/\text{mL}\right)\;\text{of reference compound}}{\text{M IC}\;\left(\text{mg}/\text{mL}\right)\;\text{of tested compound}}\\ \;\times 100 \end{array}$$

**Figure 5:**
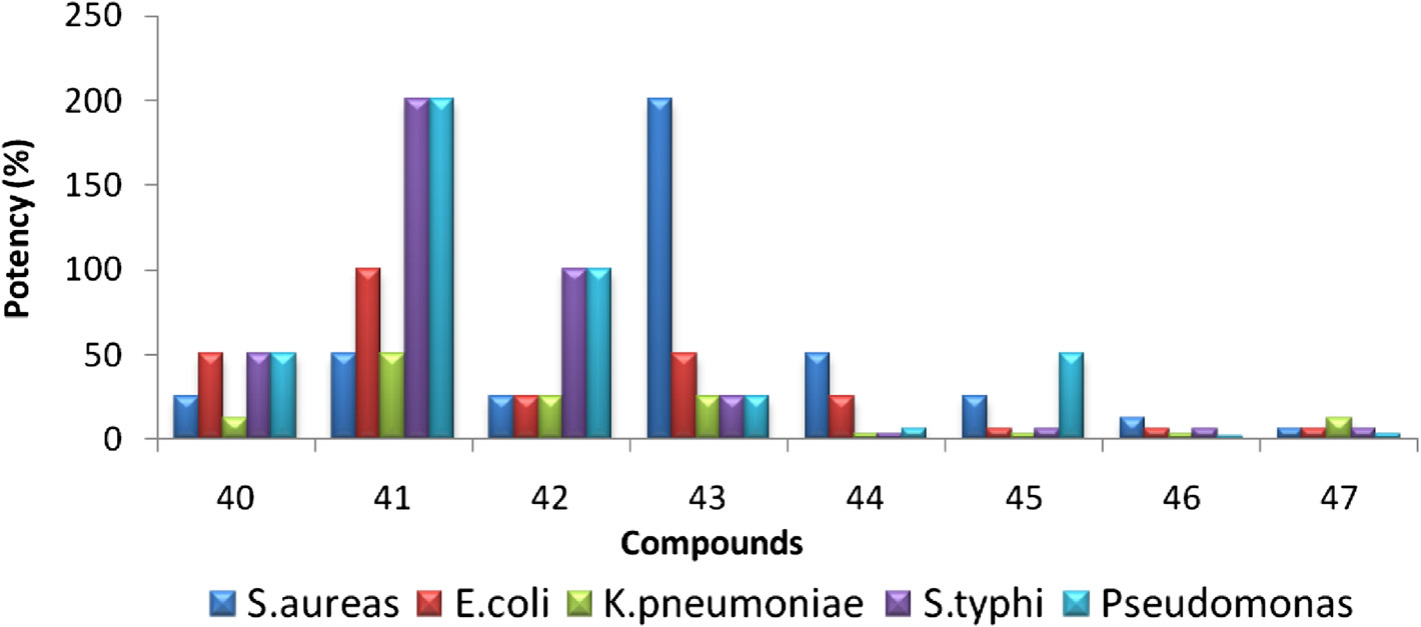
Comparison of potency of pyrazolines with ciprofloxacin (standard) against bacterial strains from serial dilution method.

**Figure 6:**
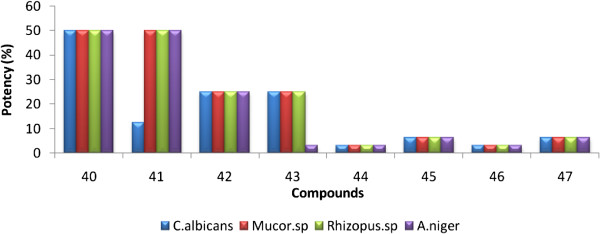
Comparison of potency of pyrazolines with ciprofloxacin (standard) against fungal strains from serial dilution method.

To comprehend it well, the results obtained from the above equation are presented as bar graphs and are shown in Figures 5 and 6.

On comparison with ciprofloxacin compounds 1–4 showed the potency against all the bacterial strains in the range of 25–100%. But, the remaining compounds exhibited lesser potency in the range of 1.5 - 50%.

With reference to ketoconazole, an equal potency is noted for compounds 1 (50%), 3(25%), 5 and 7 (3.13%), 6 and 8 (6.25%) against all the fungal strains. But 12.5% potency is noted for compound 2 against C.albicans and 3.13% is noted for compound 4 against A. niger.

## Experimental

### Preparation and characterization of catalyst

In a 50-mL Borosil beaker (Borosil Glass Works Limited, Mumbai, India), 1 g of fly ash and 0.8 mL (0.5 mol) of sulphuric acid were taken and mixed thoroughly with glass rod. This mixture was heated on a hot air oven at 85°C for 1 h, cooled to room temperature, stored in a Borosil bottle, and tightly capped [42]. This was characterized by infrared spectra and scanning electron microscopy (SEM) analysis.

Infrared spectral data of fly ash: H_2_SO_4_ is ν (per centimeter) with values 3,456(OH); 3,010 (C-H); 1,495, 1,390(C-S); 1,336, 1,154(S = O) and *op* modes 1,136, 1,090, 976, 890, 850, 820, 667, 658, 620, 580, 498, and 425.

**Figure 7:**
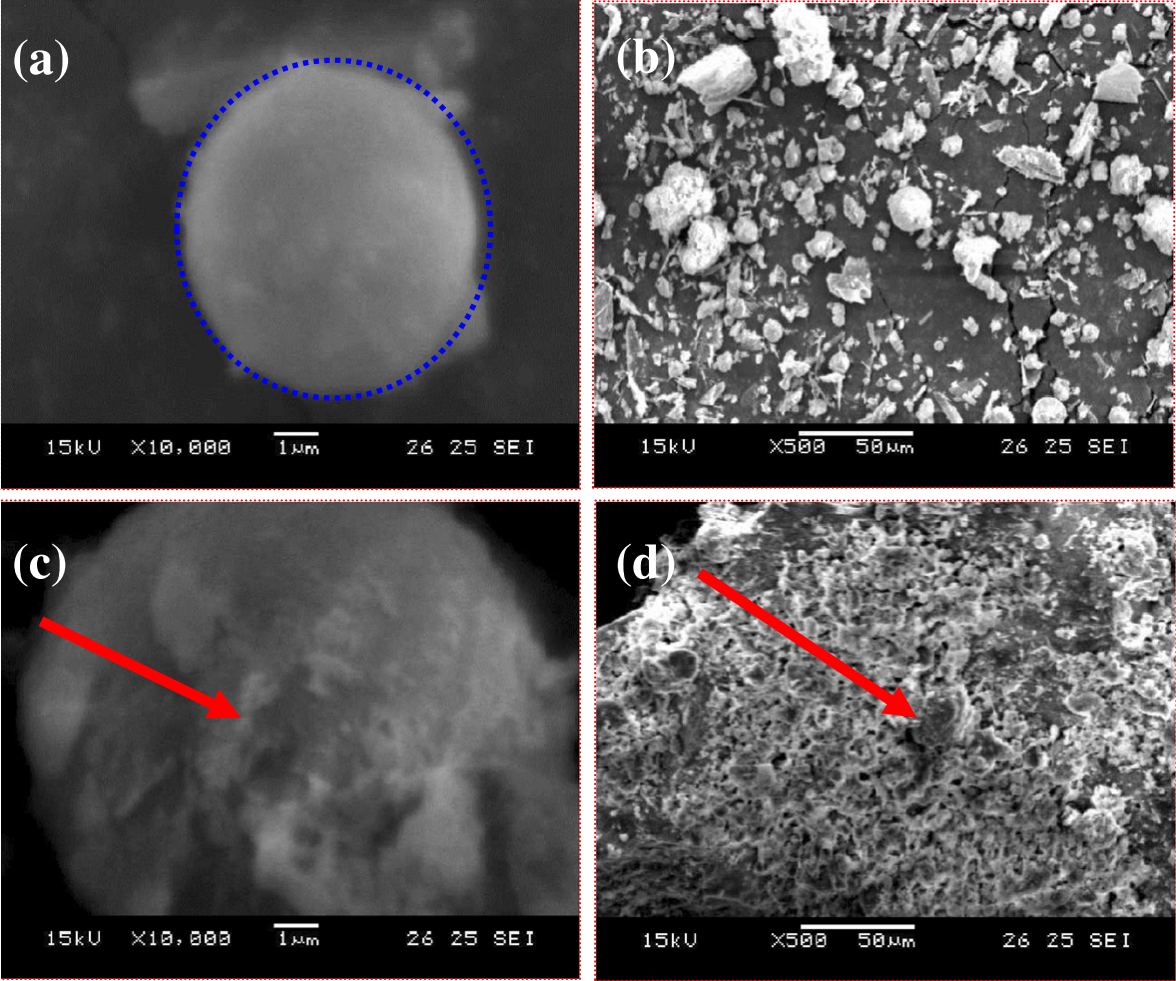
**SEM images of pure fly ash and fly ash:H2SO4.** (**a**) Pure fly ash (1 μm), (**b**) pure fly ash (50 μm), (**c**) fly ash:H_2_SO_4_ (1 μm) (red bold arrow, corroded), and (**d**) fly ash:H_2_SO_4_ (50 μm) (red bold arrow, corroded).

The SEM images of pure fly ash and fly ash:H_2_SO_4_ at two different magnifications are shown in Figure 7. Figure 1a,b depicted that there is more crystallinity found in pure fly ash. The spherical-shaped particles are clearly seen at both magnifications in Figure 7a,b. Figure 7a reveals the globular structure of pure fly ash (round-shaped particle). This is also seen in Figure 7c,d that some of the particles are slightly corroded by H_2_SO_4_ (indicated by arrow mark), and this may be due to dissolution of fly ash by H_2_SO_4_. This will further be confirmed by Figure 7d, the well-shaped particles of pure fly ash. Figure 7b is aggregated to Figure 7d due to the presence of H_2_SO_4_.

### Synthesis of substituted 5-bromo-2-thienyl chalcones

All substituted styryl 5-bromo-2-thienyl ketones were synthesized using the literature procedure [43].

### Synthesis of 1-phenyl-3(5-bromothiophen-2-yl)-5-(substituted phenyl)-2-pyrazolines

**Scheme 1:**
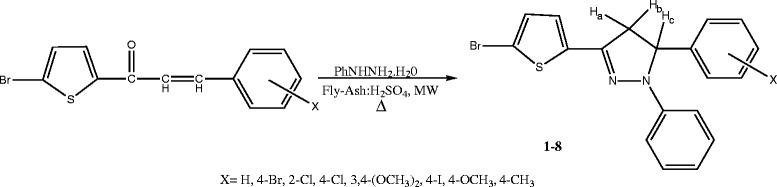
Synthesis of 1-phenyl-3(5-bromothiophen-2-yl)-5-(substituted phenyl)-2-pyrazolines.

An appropriate equi-molar quantity of 5-bromo-2-thienyl chalcones, phenylhydrazine hydrochloride (0.2 mmol), and 0.5 g of fly ash:H_2_SO_4_ was subjected to microwave irradiation for 5 to 6 min in a microwave oven (Scheme 1) (LG Grill, Intellowave, Microwave Oven, LG Electronics, Seoul, South Korea; 160 to 800 W) and then cooled to room temperature. The organic layer was separated with dichloromethane, and the solid product was obtained on evaporation. On recrystallization with benzene-hexane mixture, it gave glittering pale yellow solid. The insoluble catalyst was recycled by washing the solid reagent that remained on the filter by ethyl acetate (8 mL), followed by drying in an oven at 100°C for 1 h, and it was made reusable for further reactions. The analytical and mass spectral data are given in Table 1. Infrared and NMR spectral data of pyrazolines are presented in Table 2. The individual proton and carbon signals were unambiguously assigned by HOMOCOSY and HETCOSY spectral analysis. The ^1^ H-^1^ H COSY and ^1^ H-^13^ C COSY spectral data are presented in Table 3.

### Antibacterial activities

#### Preparation of test inoculum subculture (preparation of seeded broth)

The strains of *S. aureus*, *E. coli*, *K. pneumoniae*, *Pseudomonas* species, and *S. typhi* were inoculated in conical flasks containing 100 mL of sterile nutrient broth. These conical flasks were incubated at 37 ± 1°C for 24 h. This was referred to as the seeded broth.

#### Standardization of seeded broth (viable count) dilutions

One mL of 24-h seeded broth of each strain was diluted with 99 mL of sterile normal saline containing 0.05% Tween 80 (drops of Tween 80 in 1,000 mL normal saline). From that, 1 mL is further diluted to 10 mL sterile normal saline. This was continued to 10^−2^, 10^−3^, 10^−4^, 10^−5^, until 10^−10^ dilution of seeded broth was obtained.

#### Incubation of nutrient agar petri dishes

The dilutions were studied by inoculating 0.2 mL of each dilution onto the solidified nutrient agar medium by spread plate method, after incubation at 37 ± 1°C for 24 h. The numbers of well-formed colonies on the plates were counted. The seeded broth was then suitably diluted to have been 10^5^ to 10^7^ microorganisms per milliliter or colony-forming per milliliter. This was designated as the working stock and used for the antibacterial studies.

#### Preparation of solution of test compounds

The solution of test compounds were prepared by dissolving the same in DMSO in a specific growth bottle and stored in a refrigerator. The solution was removed from the refrigerator 1 h prior to its use and allowed to warm up to room temperature. The test compounds were prepared at a concentration of 200 μg/mL. Similarly, the standard drug solution of ciprofloxacin and ketoconazole were used respectively at a concentration of 200 μg/mL for finding the MIC.

#### Preparation of culture media

The media used for the growth of bacteria were nutrient agar medium and nutrient broth medium. The media were sterilized by autoclaving at a pressure of 15 lbs at 121°C for 20 min.

##### Nutrient agar medium

The nutrient agar medium was prepared by dissolving 28 g of nutrient agar (Hi-media, Mumbai, India) in 1,000 mL of distilled water. The following formula was followed for the preparation of nutrient agar medium: peptone, 1%; NaCl, 0.5%; beef extract, 1%; agar, 2%, and pH, 7.4 ± 0.2.

##### Nutrient broth medium

The nutrient broth medium was prepared by dissolving 13 g of nutrient broth (Hi-media, Mumbai) in 1000 mL of distilled water. The following formula was followed for preparation of nutrient broth medium: peptone 1%; NaCl, 0.5%; beef extract, 1%; agar, 1%; pH, 7.4 ± 0.2.

### Antifungal activity

#### Preparation of culture media SDA medium

The following formula was followed for the preparation of SDA medium: dextrose, 40 g; peptone, 10 g; agar, 15 g; distilled water, 1,000 mL, and pH, 5.4.

#### SDB broth

The following formula was followed for the preparation of SDB broth: dextrose, 40 g; peptone, 10 g; agar, 15 g; distilled water, 1,000 mL; and pH, 5.4.

## Conclusions

We have developed an efficient catalytic method for synthesis of 1-phenyl-3(5-bromothiophen-2-yl)-5-(substituted phenyl)-2-pyrazolines by cyclization of substituted styryl 5-bromo-2-thienyl ketones and phenyl hydrazine hydrochloride using a solvent-free environmentally greener catalyst fly ash: H_2_SO_4_ under microwave irradiation. This reaction protocol offers a simple, economical, environment friendly, non-hazardous, and easier work-up procedure and good yields. All synthesized pyrazoline derivatives showed moderate antimicrobial activities against the strains used.

## Competing interests

The authors declare that they have no competing interests.
